# Supplementary material for: Heterodimensional Kondo superlattices with strong anisotropy
Source: Nat Commun. 2024 Jun 28;15:5491. doi: 10.1038/s41467-024-49618-x (PMC11214635; doi:10.1038/s41467-024-49618-x)
Supplement: Supplementary file 1 — Supplementary Information [file 41467_2024_49618_MOESM1_ESM.pdf]

Heterodimensional Kondo superlattices with strong anisotropy

Qi Feng[1], Junxi Duan[1,*], Ping Wang[1], Wei Jiang[1,*], Huimin Peng[1], Jinrui Zhong[1], Jin Cao[1], Yuqing Hu[1], Qiuli Li[1], Qinsheng Wang, Jiadong Zhou[1,*], Yugui Yao[1,*]

[1]Key Laboratory of Advanced Optoelectronic Quantum Architecture and Measurement (MOE), School of Physics, Beijing Institute of Technology, Beijing, China

[*] Corresponding authors:

junxi.duan@bit.edu.cn

wjiang@bit.edu.cn

jdzhou@bit.edu.cn

ygyao@bit.edu.cn

Supplementary Information

Supplementary Note 1: Difference of the Kondo effect in different devices

The Kondo effect in our VS_2-VS superlattice sample is intrinsic. However, the two devices shown in the main text have different Kondo temperatures, and different field dependence. The differences can be ascribed to the difference of charge carrier density in the two devices.

According to theory, the Kondo temperature is $T_K \sim D_0\sqrt{2J\rho}e^{-\frac{1}{2J\rho}}$, where J denotes the coupling between the itinerant electrons and the local moments, ρ the density of states of the electrons at the Fermi level[1]. From the formula, T_K becomes larger when ρ is increased. In our current case, device N2 has a higher carrier density, 5×10^{22} cm^{-3}, than N1, 8.1×10^{21} cm^{-3}, which means a larger density of states. Therefore, it has a higher Kondo temperature comparing to device N1. Additionally, we also measured another Hall-bar device N3, shown in Supplementary Fig.1a. Its carrier density should be very close to device N1 since they are from the same piece of substrate grown in the same batch. Supplementary Fig. 1b shows a clear resistance upturn when the temperature decreases, and the solid line is the fitting results to Eq.(1). The extracted Kondo temperature is T_K=7 K, which is close to the one in device N1.

The Kondo temperature is extracted from fitting the resistance vs. temperature curve (*RT* curve). It is different from the temperature corresponding to the minimum of the *RT* curve. The temperature corresponding to the minimum of the *RT* curve, T_m, is determined by the competition between the Kondo effect and the scattering effect that it is more sample dependent.

T_K is the only scale governing the physics of the Kondo effect at low temperatures. Therefore, the different field response between the two devices can also be understood from the difference of the Kondo temperature. The magnetic field will split the Kondo resonance and destroy the Kondo effect. A critical field H_c, $H_c(T=0) \sim 0.5k_BT_K$, is defined to characterize the effect, which agrees quite well with our measurement[2]. For $T_K \sim 7$ K, $H_c(T=0) \sim 2.59\ T$. At finite temperature, $H_c \approx H_c(T=0)$ if $T < 0.25\,T_K$ but becomes smaller at higher temperature. The formula also shows that the Kondo effect in samples with higher Kondo temperatures can survive under higher magnetic fields. In our case, device N1 has a lower Kondo temperature than device N2. Therefore, the Kondo effect in N1 can be destroyed by a lower field comparing

to N2.

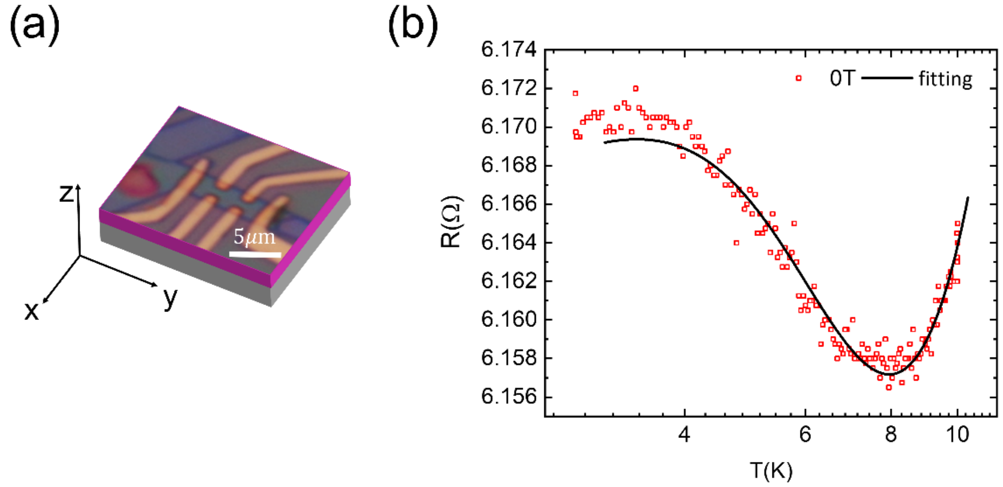

Supplementary Fig. 1. The Kondo effect in device N3. (a) The optical image of device N3. (b) Semilogarithmic plots of the temperature dependence of resistance of device N3. The solid line is the fitting of Eq. (1) with the Langevin function.

Supplementary Note 2: Magnetic anisotropy

To corroborate the unique effect of the 1D VS chains, we carry out first-principles calculations of the VS_2-VS heterodimensional superlattice. Different magnetic configurations are calculated to study the the magnetic anisotropy of the magnetic ground states. Supplementary Fig. 2 shows the magnetocrystalline anisotropy energy (MAE) for the antiferromagnetic (AFM) phase. Supplementary Fig. 3 shows the MAE for the ferromagnetic (FM) phase.

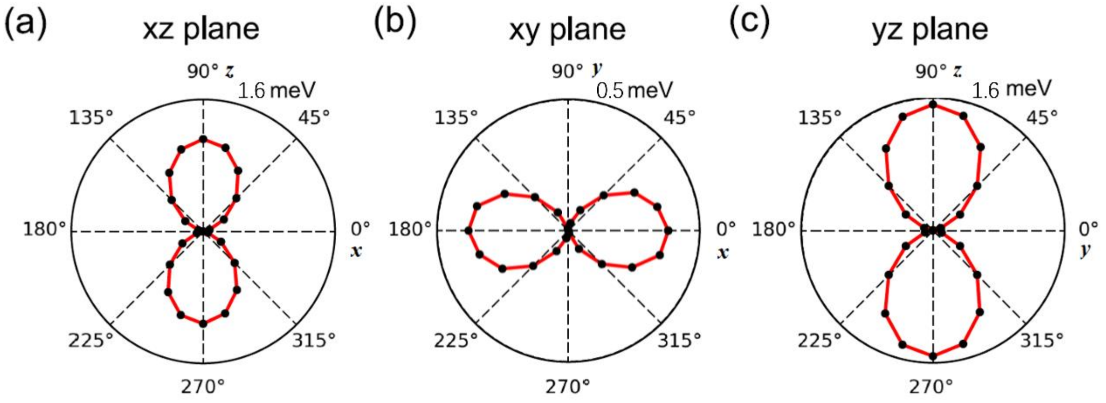

Supplementary Fig. 2. Magnetocrystalline anisotropy energy (MAE) for the AFM phase. (a) MAE in xz plane. (b) MAE in xy plane. (c) MAE in yz plane. The MAE is about 0.5 meV per unit cell (3×10^3J/m^3), with the magnetization easy axis along the *y* axis.

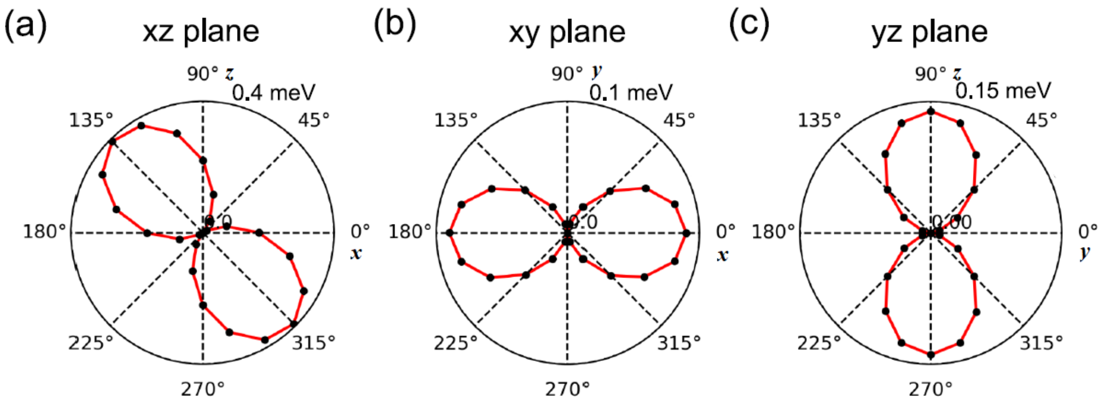

Supplementary Fig.3. Magnetocrystalline anisotropy energy (MAE) for the FM phase. (a) MAE in xz plane. (b) MAE in xy plane. (c) MAE in yz plane. The MAE is about 0.1 meV per unit cell (6×10^4J/m^3), with the magnetization easy axis along the *y* axis.

Reference

1. Hewson AC. *The Kondo Problem to Heavy Fermions*. Cambridge University Press (1993).

2. Costi TA. Kondo effect in a magnetic field and the magnetoresistivity of kondo alloys. *Phys. Rev. Lett.* **85**, 1504-1507 (2000).
